# Group Structures and Derivations on *PMS*-algebras

**DOI:** 10.12688/f1000research.159712.1

**Published:** 2025-01-13

**Authors:** Nibret Melese Kassahun, Berhanu Assaye Alaba, Yohannes Gedamu Wondifraw, Zelalem Teshome Wale

**Affiliations:** 1 Mathematics, Bahir Dar University, Bahir Dar, Amhara, 6000, Ethiopia; 2 Mathematics, Debre Markos University, Debre Markos, Amhara, None, Ethiopia; 3 Mathematics, Addis Ababa University, Addis Ababa, Addis Ababa, 1000, Ethiopia

**Keywords:** P MS-algebras, derivations, generalized derivation, torsion free P MS-algebra, semigroup

## Abstract

**Background:**

PMS-algebras are a specific algebraic structure that generalizes a propositional algebra called BCK-algebra. This paper delves into the intricate group structure of these algebras and the concept of derivations within this framework.

**Methods:**

We employ rigorous mathematical techniques to analyze the properties of derivations in P MS-algebras. This involves examining various characteristics of derivations and investigating their behavior in specific subcategories, such as torsion-free P MS-algebras.

**Results:**

Our research reveals several key findings. Firstly, we establish that the set of all derivations associated with the binary operation defined on a P MS-algebra constitutes a semigroup. Secondly, we provide a comprehensive analysis of generalized derivations, d-invariant ideals, fixed sets, and torsion-free P MS-algebras within the context of P MS-algebras.

**Conclusions:**

This study contributes to a deeper understanding of the algebraic structure of P MS-algebras and the role of derivations within this context. The findings presented here have implications for further research in abstract algebra and related fields.

## 1. Introduction

Iseki ^1^ introduced a type of abstract algebra known as BCK-algebras. In a subse- quent publication, Iseki ^2^ introduced another class of algebra called BCI-algebras, which can be seen as a generalization of BCK-algebras. Iseki studied some properties of BCI-algebras. Tamalibrasi and Megalai ^3^ introduced T M-algebras based on propositional calculus. Mostafa et al. ^4^ introduced the concepts of KUS-algebras, KUS-ideals, and KUS-subalgebras, and investigated their relationships. Selvam and Nagalakshmi ^5^ introduced an algebraic structure called PMS-algebra, which generalizes the notions of BCK/BCI/TM/KUS-algebras. They also explored additional properties of PMS-algebras and their ideals.

Derivations are an important research area in algebraic structure theory, developed from the concepts of Galois theory and invariant theory. Derivations are a crucial research area in the theory of algebraic structures in mathematics. The ring with derivation is an ancient concept that significantly integrates analysis, algebraic geometry, and algebra. Jun and Xin ^6^ extended the concept of derivations in ring and near-ring theory to BCI algebras and related properties. Ganeshkumar and Chandramouleeswaran ^7^ derivations and generalized derivations on TM algebras were studied. Also, Kim and Lee ^8^ introduced the notion of derivations on BE algebras and studied their properties. Furthermore, Jana et al. ^9^ introduced derivations, generalized, and f-derivations and characterized their properties on KUS algebras. Sawika et al. ^10^ studied the notions of (l, r)-derivations and derivations of UP-algebras, and their properties were investigated. Recently, by Rong et al., ^11^ the properties of derivations on FI-algebras and the relationship between derivations and ideals were investigated. Sugianti et al. ^12^ introduced the notion of a (l, r)-derivation, a (r, l)-derivation, and derivation in BM-algebras and investigated related properties. Ganesan and Kandaraj ^13^ studied the idea of generalized derivations of BH-algebras and characterized new properties based on the context of BH-algebras in their work. Additionally, Muangkarn et al. ^14^ studied the derivations of d-algebras based on endomorphisms.

This paper focuses on introducing the fundamental concepts of derivations in PMS-algebras, exploring the properties of generalized derivations, and providing a definition of torsion-free PMS-algebras. The study also investigates the properties of a d-invariant ideal and establishes conditions for its existence.

## 2. Methods

In this section, we will address the concepts and basic properties of PMS-algebras that we need for the main results in the next sections. These concepts are taken from Selvam and Nagalakshmi. ^5^

Definition 1. ^5^ A PMS-algebra is an algebra (X; ⋆, 0) of type (2, 0) with a constant “0” and a binary operation “⋆” satisfying the following axioms:

1. 0 ⋆ a = a;
2. (b ⋆ a) ⋆ (c ⋆ a) = c ⋆ b for all a, b, c ∈ X.

In X, we define a binary relation “≤” by a ≤ b if and only if a ⋆ b = 0.

Proposition 1. ^5^ In a PMS-algebra (X; ⋆, 0) the following properties hold for all a, b, c ∈ X.

1. a ⋆ a = 0.
2. (b ⋆ a) ⋆ c = (c ⋆ a) ⋆ b.
3. (b ⋆ a) ⋆ a = b.
4. a ⋆ (b ⋆ a) = b ⋆ 0.
5. If a ⋆ b = 0 and b ⋆ a = 0, then a = b.

Definition 2. ^5^ Let S be a nonempty subset of a PMS-algebra (X; ⋆, 0). Then S is called a PMS-subalgebra of X if a*b ∈ S for all a, b ∈ S.

Example 1. ^5^ Consider (Z; ⋆, 0) where a ⋆ b = b - a for all a, b ∈ X. Then (Z; ⋆, 0) is a PMS-algebra, and the set E of even integers is a PMS-subalgebra of Z.

Definition 3. ^5^ Let X be a PMS-algebra and I be a subset of X. Then I is called a PMS-ideal of X if

1. 0 ∈ I
2. c ⋆ b ∈ I and c ⋆ a ∈ I ⇒ b ⋆ a ∈ I for all a, b, c ∈ X.

Definition 4. ^5^ Let X be a PMS-algebra. The set G(X)={a∈X|a⋆0=a} is called the G-part of X.

Definition 5. Let X be a PMS-algebra. The set B(X)={a∈X|a⋆0=0} is called the p-radical of X.

Definition 6. ^5^ A PMS-algebra X is said to be Medial if (a ⋆ b) ⋆ (c ⋆ d) = (a ⋆ c) ⋆ (b ⋆ d) for all a, b, c, d ∈ X.

## 3. Group structure and Derivations on P MS-algebras

In this section, we will obtain an abelian group from a PMS-algebra by defining binary operations on it. Moreover, we introduce derivations and similarly the basic properties of derivations on PMS-algebras.

Remark 1. Let (X; ⋆, 0) be a PMS-algebra. If b ⋆ a = c ⋆ a, then b = c for all a, b, c ∈ X.

Proof. Let b ⋆ a = c ⋆ a.

Now b = 0 ⋆ b = (a ⋆ a) ⋆ b = (b ⋆ a) ⋆ a = (c ⋆ a) ⋆ a = (a ⋆ a) ⋆ c = 0 ⋆ c = c.

Theorem 2. Let (X, ⋆, 0) be a PMS-algebra and define ′ ^′^+ ^′′^ as a + b = (b ⋆ 0) ⋆ a, ∀a, b ∈ X. Then the following holds:

1. a + 0 = a = 0 + a.
2. Addition is associative.
3. If a + b = 0, then a = b ⋆ 0.
4. Addition is commutative.
5. The additive inverse of a is a ⋆ 0.

Proof.

1. a + 0 = (0 ⋆ 0) ⋆ a = 0 ⋆ a = a and 0 + a = (a ⋆ 0) ⋆ 0 = (0 ⋆ 0) ⋆ a = 0 ⋆ a = a.
Hence a + 0 = a = 0 + a.
2. By applying the definition of “+” and by simplifying, we get the result.
3. a + b = 0 ⇒ (b ⋆ 0) ⋆ a = a ⋆ a, by applying right cancellation we get a = b ⋆ 0.
4. a + b = (b ⋆ 0) ⋆ a = (a ⋆ 0) ⋆ b = b + a.
5. a + (a ⋆ 0) = ((a ⋆ 0) ⋆ 0) ⋆ a = (a ⋆ 0) ⋆ (a ⋆ 0) = a ⋆ a = 0.

Hence a ⋆ 0 is the inverse of a.

Definition 7. Let (X; ⋆, 0) be a PMS-algebra. Define a + b = (b⋆0) ⋆ a and -a = (a⋆0). Then (X, +) is an abelian group.

Remark 2. If we have a PMS-algebra (X, ⋆, 0) it follows from the above definition that (X, +) is an abelian group with -b = b ⋆ 0, ∀ b ∈ X. Then we have b - a = a ⋆ b, ∀ a, b ∈ X. On the other hand if we have an abelian group (X, +) with an identity “0” and define a ⋆ b = b - a, we get a PMS-algebra (X, ⋆, 0), where a + b = (b ⋆ 0) ⋆ a, ∀ a, b ∈ X.

Example 2. Let (X, ⋆, 0) be a PMS-algebra as shown in Cayley Table 1.

**Table 1.** PMS-algebra.

⋆	*0*	*1*	*2*	*3*	* 4*
*0*	*0*	*1*	*2*	*3*	*4*
*1*	*4*	*0*	*1*	*2*	*3*
*2*	*3*	*4*	*0*	*1*	*2*
*3*	*2*	*3*	*4*	*0*	*1*
*4*	*1*	*2*	*3*	*4*	*0*

Define a + b = (b ⋆ 0) ⋆ a

Then (X, +) is an abelian group as shown in Table 2.

**Table 2.** Abelian group.

*+*	*0*	*1*	*2*	*3*	* 4*
*0*	*0*	*1*	*2*	*3*	*4*
*1*	*1*	*2*	*3*	*4*	*0*
*2*	*2*	*3*	*4*	*0*	*1*
*3*	*3*	*4*	*0*	*1*	*2*
*4*	*4*	*0*	*1*	*2*	*3*

Definition 8. Let (X; ⋆, 0) be a PMS-algebra. Define a binary operation“∧” on X by x ∧ y = (x ⋆ y) ⋆ y for all x, y ∈ X.

Definition 9. Let (X; ⋆, 0) be a PMS-algebra. A self-map d: X → X is said to be a (left, right)-derivation of X, denoted by, (l, r)-derivation if d(x ⋆ y) = (d(x) ⋆ y) ∧ (x ⋆ d(y)) for all x, y ∈ X.

Example 3. Consider the PMS-algebra X = {0, 1, 2, 3} as shown in Table 3:

**Table 3.** (l, r)-derivation on X.

⋆	*0*	*1*	*2*	* 3*
*0*	*0*	*1*	*2*	*3*
*1*	*1*	*0*	*3*	*2*
*2*	*2*	*3*	*0*	*1*
*3*	*3*	*2*	*1*	*0*

Then the self-map d: X → X defined by d(0) = 3, d(1) = 2, d(2) = 1 and d(3) = 0 is a (l, r)-derivation on X.

Remark 3. Let d: X → X be a (l, r)-derivation on X. Then d(x ⋆ y) = d(x) ⋆ y for all x, y ∈ X.

Definition 10. Let (X; ⋆, 0) be a PMS-algebra. A self-map d: X → X is said to be a (right, left)-derivation of X, denoted by, (r, l)-derivation if d(x ⋆ y) = (x ⋆ d(y)) ∧ (d(x) ⋆ y) for all x, y ∈ X.

Example 4. Let X be the PMS-algebra defined in the above example 3, the self-map d: X → X defined by d(0) = 1, d(1) = 0, d(2) = 3 and d(3) = 2 is a (r, l)-derivation on X.

Remark 4. Let d: X → X be a (r, l)-derivation on X. Then d(x ⋆ y) = x ⋆ d(y) for all x, y ∈ X.

Definition 11. Let d: X → X be a self-map on a PMS-algebra (X; ⋆, 0). Then the self-map d is said to be a derivation on X if d is both a (l, r)-derivation and a (r, l)-derivation on X.

Example 5. Let (Z; ⋆, 0) be a PMS-algebra with x ⋆ y = y − x. Then the map d: Z → Z defined

1. by d(x) = x is a derivation on Z.
2. by d(x) = x-1 is a (r, l) derivation on Z but not a (l, r)-derivation. Hence, d is not a derivation.

Remark 5. Let d: X→ X be a derivation on X. Then d(x ⋆ y) = d(x) ⋆y = x ⋆ d(y) for all x, y ∈ X.

Theorem 3. Let (X; ⋆, 0) be a PMS-algebra, and d: X→ X be a (l, r) -derivation. If y ≤ x, then d(x) = d(y) for all x, y ∈ X.

Proof. Let y ≤ x. Then y ⋆ x = 0.

Now

$$\begin{array}{l} d\left(x\right)=d\left(0⋆x\right)\\ \;=d\left(\left(y⋆x\right)⋆x\right)\\ \;=d\left(\left(y⋆x\right)⋆\left(0⋆x\right)\right)\\ \;=d\left(0⋆y\right)\\ \;=d\left(y\right) \end{array}$$

Hence, d(x) = d(y).

Definition 12. Let (X; ⋆, 0) be a PMS-algebra. A self-map d on X is said to be regular if d(0) = 0.

Example 6. Let X be the PMS-algebra defined as in Example **3**. The self-map d: X→ X defined by d(0) = 0, d(1) = 1, d(2) = 2, d(3) = 3 is a regular derivation.

Theorem 4. Let (X; ⋆, 0) be a PMS-algebra. If d: X→ X is a regular (l, r)-derivation, then d(x) ≤ x for all x ∈ X.

Proof. Suppose d is a regular (l, r)-derivation on X. Then d(0) = d(x ⋆ x) implies d(x) ⋆ x = 0. Thus d(x) ≤ x.

Theorem 5. Let (X, ⋆, 0) be a PMS-algebra. If d: X→ X be a regular (r, l)-derivation, then x ≤ d(x) for all x ∈ X.

Proof. Suppose d is a regular (r, l)-derivation on X. Then d(0) = d(x ⋆ x) implies that x ⋆ d(x) = 0. Thus x ≤ d(x).

Theorem 6. Let (X; ⋆, 0) a PMS-algebra and d: X → X be a (l, r)-derivation. Then d is regular if and only if d(x) = x.

Proof. Let d be a derivation on X. Suppose that d(0) = 0.

Now

$$\begin{array}{l} d\left(x\right)=d\left(0⋆x\right)\\ \;=\left(d\left(0\right)⋆x\right)\wedge \left(0⋆d\left(x\right)\right)\\ \;=\left(0⋆x\right)\wedge \left(d\left(x\right)\right)\\ \;=x\wedge d\left(x\right)\\ \;=\left(x⋆d\left(x\right)\right)⋆d\left(x\right)\\ \;=\left(d\left(x\right)⋆d\left(x\right)\right)⋆x\\ \;=0⋆x\\ \;=x. \end{array}$$

Therefore, d(x) = x.

Conversely, assume that d(x) = x, ∀ x ∈ X.

In particular, d(0) = 0. Hence d is regular.

Theorem 7. Let G(X) be the G-part of a PMS-algebra X and d be a (l, r)-derivation on X. Then d(x) ∈ G(X), ∀x ∈ G(X).

Proof. Let x∈G(X). Then x ⋆ 0 = x. Now

$$\begin{array}{l} d\left(x\right)=d\left(x⋆0\right)\\ \;=d\left(x\right)⋆0. \end{array}$$

Hence d(x) ∈ G(X).

Theorem 8. Let B(X) be the P-radical of a PMS-algebra X and d be a (l, r)-derivation on B(X). Then d is regular.

Proof. Let x ∈ B(X). Then x ⋆ 0 = 0.

Now

$$\begin{array}{l} d\left(0\right)=d\left(x⋆0\right)\\ \;=d\left(x\right)⋆0\\ \;=0 \end{array}$$

Hence d(0) = 0:

Therefore, d is a regular derivation on B(X).

Theorem 9. Let (X; ⋆, 0) be a PMS-algebra and d: X → X be a (l, r)-derivation. Then

1. d(x) ⋆ x = 0 ∀x ∈ X if and only if d is regular.
2. d(x ⋆ d(x)) = 0 ∀x ∈ X.

Proof.

1. Suppose that d(x) ⋆ x = 0. Now
$$\begin{array}{l} d\left(0\right)=d\left(x⋆x\right)\\ \;=d\left(x\right)⋆x\\ \;=0 \end{array}$$
Hence, d is regular.
Conversely, assume that d(0) = 0
Now d(x ⋆ x) = 0 implies that d(x) ⋆ x = 0 Hence d(x) ⋆ x = 0.
2. d(x ⋆ d(x)) = d(x) ⋆ d(x) = 0.

Theorem 10. Let (X; ⋆, 0) be a PMS-algebra and d: X → X be a (r, l)-derivation. Then

1. x ⋆ d(x) = 0, ∀x ∈ X if and only if d is regular.
2. d(d(x) ⋆ x) = 0, ∀x ∈ X.

Proof.

1. Suppose that d(x) ⋆ x = 0 Now
$$\begin{array}{l} d\left(0\right)=d\left(x⋆x\right)\\ \;=d\left(x\right)⋆x\\ \;=0 \end{array}$$
Hence, d is regular.
Conversely, assume that d(0) = 0
Now d(x ⋆ x) = d(0) implies that d(x) ⋆ x = 0
Hence d(x) ⋆ x = 0.
2. d(d(x) ⋆ x) = d(x) ⋆ d(x) = 0.

Theorem 11. Let d be a self-map of a PMS-algebra X.

1. Let d be a (l, r) -derivation on X. Then d regular if and only if d(x) = x ∧ d(x).
2. Let d be a (r, l) -derivation on X. If d is regular, then d(x) = d(x) ∧ x.

Proof.

1. Let d be a self-map on a PMS-algebra. Suppose that d is regular (l, r)-derivation on X.
$$\begin{array}{l} d\left(x\right)=d\left(0⋆x\right)\\ \;=\left(d\left(0\right)⋆x\right)\wedge \left(0⋆d\left(x\right)\right)\\ \;=\left(0⋆x\right)\wedge \left(0⋆d\left(x\right)\right)\\ \;=x\wedge d\left(x\right). \end{array}$$
Conversely, assume that d(x) = x∧d(x). Then d(0) = 0∧d(0) = (0 ⋆ d(0)) ⋆ d(0) = (d(0) ⋆ d(0)) ⋆ 0 = 0 ⋆ 0 = 0.
2. Let d be regular (l, r)-derivation.
$$\begin{array}{l} d\left(x\right)=d\left(0⋆x\right)\\ \;=\left(0⋆d\left(x\right)\right)\wedge \left(d\left(0\right)⋆x\right)\\ \;=\left(0⋆d\left(x\right)\right)\wedge \left(0⋆x\right)\\ \;=d\left(x\right)\wedge x. \end{array}$$

Theorem 12. Let d be a (r, l)-derivation on a PMS-algebra X. Then

1. d(0) = x ⋆ d(x).
2. d is 1-1.
3. If there is an element x∈X such that d(x) = x, then d is the identity map.
4. If there is an element x∈X such that d(y) ⋆ x = 0 for all y∈X, then d(y) = x, that is, d is a constant map.

Proof.

1. d(0) = d(x ⋆ x) = x ⋆ d(x) Because d is a (r, l)-derivation on X.
2. Let x, y ∈ X and d(x) = d(y).
d(0)=d(x⋆x)=x⋆d(x) (1)
d(0)=d(y⋆y)=y⋆d(y) (2)
From (1) and (2) we have by x ⋆ d(x) = y ⋆ d(y) implies that x ⋆ d(x) = y ⋆ d(x). Hence by right cancellation we have x = y.
Therefore d is a one to one map.
3. Let x ∈ X such that d(x) = x.
Let y ∈ X be any element of X. Then y = 0 ⋆ y = (x ⋆ x) ⋆ y = (y ⋆ x) ⋆ x. Then
$$\begin{array}{l} d\left(y\right)=d\left(\left(y⋆x\right)⋆x\right)\\ \;=\left(y⋆x\right)⋆d\left(x\right)\\ \;=\left(y⋆x\right)⋆x\\ \;=y \end{array}$$
Hence, d(x) = x, ∀x∈X.
This proves that d is the identity map on X.
4. Given d(y) ⋆ x = 0 implies that d(y) ⋆ x = x ⋆ x, and hence by right cancellation we have d(y) = x.

Corollary 1. Let (X; ⋆, 0) be a PMS-algebra. If d is a (r, l)-derivation on X, then x ⋆ d(x) = y ⋆ d(y) ∀x, y ∈ X.

Proof. Let x, y ∈ X. Then

d(0)=d(x⋆x)=x⋆d(x) (3)

d(0)=d(y⋆y)=y⋆d(y) (4)

From (3) and (4) we have x ⋆ d(x) = y ⋆ d(y)

Theorem 13. Let X be a PMS-algebra and d be a regular (l, r)-derivation on X. Then Ker d = {x ∈ X: d(x) = 0} is a PMS-subalgebra of X.

Proof. Since d is a regular, d(0) = 0.

Hence 0 ∈ Ker d. It follows that Ker d ≠∅ .

Let x, y ∈ Ker d. Then d(x) = 0,

d(y) = 0. Now we have

$$\begin{array}{l} d\left(x⋆y\right)=d\left(x\right)⋆y\\ \;=x⋆y\\ \;=d\left(x\right)⋆d\left(y\right)\\ \;=0⋆0\\ \;=0. \end{array}$$

Hence x ⋆ y ∈ Ker d.

Therefore Ker d is a PMS-subalgebra of X.

Theorem 14. Let d be a (r, l)-derivation on a PMS-algebra X. If x ≤ y and x ∈ Ker d, then y ∈ Ker d.

Proof. Let x≤y and x∈Ker d. Then x ⋆ y = 0 and d(x) = 0.

Now d(y) = d(0 ⋆ y) = d((x ⋆ y) ⋆ y) = d((y ⋆ y) ⋆ x) = d(0 ⋆ x) = d(x) = 0. Hence y ∈ Ker d.

Theorem 15. Let X be medial of a PMS-algebra and d be a regular (l, r)-derivation on X. Then Ker d is a PMS-ideal of X.

Proof. Let z ⋆ x ∈ Ker d and z ⋆ y ∈ Ker d. Then d(z ⋆ x) = 0 and d(z ⋆ y) = 0. Now

$$\begin{array}{l} d\left(x⋆y\right)=d\left(0⋆\left(x⋆y\right)\right)\\ \;=d\left(\left(z⋆z\right)⋆\left(x⋆y\right)\right)\\ \;=d\left(\left(z⋆x\right)⋆\left(z⋆y\right)\right)\\ \;=d\left(z⋆x\right)⋆\left(z⋆y\right)\\ \;=d\left(z⋆x\right)⋆d\left(z⋆y\right)\\ \;=0⋆0\\ \;=0. \end{array}$$

Hence x ⋆ y ∈ Ker d

Therefore Ker d is a PMS-ideal of X.

Theorem 16. Let X be a PMS-algebra and d be a (l, r)-derivation on X. Then Ker d = {0} if and only if d is regular.

Proof. Suppose that Ker d = {0}.

Let x ∈ Ker d. Then d(x) = 0, ∀x ∈ X in particular d(0) = 0. Hence d is regular.

Conversely, assume that d is regular Let x ∈ Ker d. Then d(x) = 0.

Now 0 = d(0) = d(x ⋆ x) = d(x) ⋆ x = 0 ⋆ x = x. Hence, x = 0

Thus Ker d = {0}.

Definition 13. Let X be a PMS-algebra and d: X→ X be a derivation of X. Define ${\text{Fix}}_{d}\left(X\right)=\left\{x\in X|d\left(x\right)=x\right\}$ is called the fixed set of X.

Theorem 17. Let (X; ⋆, 0) be a PMS-algebra and d be a (l, r)-derivation on X. Then Fix _d_(X) is a PMS-subalgebra of X.

Proof. Since 0 ∈ X, and d(0) = 0. Hence 0 ∈ Fix _d_(X). It follows that Fix _d_(X) ≠∅ .

Let x, y ∈ Fix _d_(X). Then d(x) = x and d(y) = y.

Now

$$\begin{array}{l} d\left(x⋆y\right)=d\left(x\right)⋆y\\ \;=x⋆d\left(y\right)\\ \;=x⋆y \end{array}$$

Hence d(x ⋆ y) = x ⋆ y implies that x ⋆ y ∈ Fix _d_(X). Therefore, Fix _d_(X) is a PMS-subalgebra of X.

Theorem 18. Let d be a (r, l)-derivation on a PMS-algebra X. If x ≤ y and y ∈ Fix _d_(X), then x ∈ Fix _d_(X).

Proof. Let x ≤ y and y ∈ Fix _d_(X). Then x ⋆ y = 0 and d(y) = y. Now

$$\begin{array}{l} d\left(x\right)=d\left(0⋆x\right)\\ \;=d\left(\left(y⋆y\right)⋆x\right)\\ \;=d\left(\left(x⋆y\right)⋆y\right)\\ \;=\left(x⋆y\right)⋆d\left(y\right)\\ \;=\left(x⋆y\right)⋆y\\ \;=\left(y⋆y\right)⋆x\\ \;=0⋆x\\ \;=x \end{array}$$

Hence, x ∈ Fix _d_(X).

Theorem 19. Let X be a medial PMS-algebra and d be a (r, l)-derivation on X. Then Fix _d_(X) is a PMS-ideal of X.

Proof.

1. Since d(x) = x for all x ∈ Fix _d_(X), d(0) = 0.
Hence, 0 ∈ Fix _d_(X).
2. Let z ⋆ x∈Fix _d_(X) and z ⋆ y∈Fix _d_(X). Then d(z ⋆ x) = z ⋆ x and d(z ⋆ y) = z ⋆ y.
Now
$$\begin{array}{l} d\left(x⋆y\right)=d\left(0⋆\left(x⋆y\right)\right)\\ \;=d\left(\left(z⋆z\right)⋆\left(x⋆y\right)\right)\\ \;=d\left(\left(z⋆x\right)⋆\left(z⋆y\right)\right)\\ \;=\left(z⋆x\right)⋆d\left(z⋆y\right)\\ \;=\left(z⋆x\right)⋆\left(z⋆y\right)\\ \;=\left(z⋆z\right)⋆\left(x⋆y\right)\\ \;=0⋆\left(x⋆y\right)\\ \;=x⋆y \end{array}$$

Hence, x ⋆ y ∈ Fix _d_(X).

Therefore, Fix _d_(X) is an ideal of X.

Theorem 20. Let X be a PMS-algebra and d be a (r, l)-derivation on X. If y≤x and d(x) = x, then d(y) = y.

Proof. d(y) = d(0 ⋆ y) = d((x ⋆ x) ⋆ y) = d((y ⋆ x) ⋆ x) = (y ⋆ x) ⋆ d(x) = (y ⋆ x) ⋆ x = (x ⋆ x) ⋆ y = 0 ⋆ y = y.

Definition 14. Let X be a PMS-algebra. A self-map d on X is said to be an isotone if x≤y implies that d(x) ≤d(y) for all x, y∈X.

Theorem 21. Let X be a PMS-algebra and d be a regular (l, r)-derivation on X. Then the following properties are equivalent:

1. d is an isotone derivation on X.
2. x ≤ y implies d(x ⋆ y) = d(x) ⋆ y.

Proof. Suppose that d is an isotone (l, r)-derivation on X. Let x, y∈X such that x≤y. Then we have d(x ⋆ y) = d(0) = 0. Since d is isotone, we get d(x) ⋆ d(y) = 0. Now

$$\begin{array}{l} d\left(x⋆y\right)=0\\ \;=d\left(x\right)⋆d\left(y\right)\\ \;=d\left(x\right)⋆d\left(0⋆y\right)\\ \;=d\left(x\right)⋆\left(d\left(0\right)⋆y\right)\\ \;=d\left(x\right)⋆\left(0⋆y\right)\\ \;=d\left(x\right)⋆y. \end{array}$$

Therefore, d(x ⋆ y) = d(x) ⋆ y.

Assume that x ≤ y implies d(x ⋆ y) = d(x) ⋆ y. Let x, y∈X such that x ≤ y. Now

$$\begin{array}{l} d\left(x\right)⋆d\left(y\right)=d\left(x\right)⋆d\left(0⋆y\right)\\ \;=d\left(x\right)⋆\left(d\left(0\right)⋆y\right)\\ \;=d\left(x\right)⋆\left(0⋆y\right)\\ \;=d\left(x\right)⋆y\\ \;=d\left(x⋆y\right)\\ \;=d\left(0\right)\\ \;=0 \end{array}$$

Hence, d(x) ⋆ d(y) = 0 implies that d(x) ≤ d(y).

Therefore, d is an isotone derivation on X.

Definition 15. Let LDer(X) be the set of all (l, r)-derivations on X. Define a binary operation “∧” on LDer(X) by (d _1_ ∧ d _2_)(x) = d _1_(x) ∧ d _2_(x) for all x ∈ X, where d _1_, d _2_ ∈ LDer(X).

Lemma 1. If d _1_ and d _2_ are (l, r)-derivations on X, then (d _1_ ∧ d _2_) is also a (l, r) derivation on X. Proof.

$$\begin{array}{l} \left(d_{1}\wedge d_{2}\right)\left(x⋆y\right)=d_{1}\left(x⋆y\right)\wedge d_{2}\left(x⋆y\right)\\ \;=\left(d_{1}\left(x\right)⋆y\right)\wedge \left(d_{2}\left(x\right)⋆y\right)\\ \;=\left(\left(d_{1}\left(x\right)⋆y\right)⋆\left(d_{2}\left(x\right)⋆y\right)\right)⋆\left(d_{2}\left(y\right)⋆y\right)\\ \;=d_{1}\left(x\right)⋆y \end{array}$$ (5)

$$\begin{array}{l} \left(d_{1}\wedge d_{2}\right)\left(x\right)⋆y=\left(d_{1}\left(x\right)\wedge d_{2}\left(x\right)\right)⋆y\\ \;=\left(\left(d_{1}\left(x\right)⋆d_{2}\left(x\right)\right)⋆d_{2}\left(x\right)\right)⋆y\\ \;=d_{1}\left(x\right)⋆y \end{array}$$ (6)

From (5) and (6) we have (d _1_ ∧ d _2_)(x ⋆ y) = (d _1_ ∧ d _2_)(x) ⋆ y. Hence, (d _1_ ∧ d _2_) (l, r)-derivation.

Lemma 2. The binary operation “∧” defined on LDer(X) is associative. Proof. Let X be a PMS-algebra.

Let d _1_, d _2_, d _3_ be (l, r)-derivations on X. Now

$$\begin{array}{l} \left(\left(d_{1}\wedge d_{2}\right)\wedge d_{3}\right)\left(x\right)=\left(\left(d_{1}\wedge d_{2}\right)\wedge d_{3}\right)\left(0⋆x\right)\\ \;=\left(d_{1}\wedge d_{2}\right)\left(0⋆x\right)\wedge d_{3}\left(0⋆x\right)\\ \;=\left(d_{1}\left(0\right)⋆x\right)\wedge \left(d_{3}\left(0\right)⋆x\right)\\ \;=\left(\left(d_{1}\left(0\right)⋆x\right)⋆\left(d_{3}\left(0\right)⋆x\right)\right)⋆\left(d_{3}\left(0\right)⋆x\right)\\ \;=d_{1}\left(0\right)⋆x \end{array}$$ (7)

$$\begin{array}{l} \left(d_{1}\wedge \left(d_{2}\wedge d_{3}\right)\right)\left(x\right)=\left(d_{1}\wedge \left(d_{2}\wedge d_{3}\right)\right)\left(0⋆x\right)\\ \;=d_{1}\left(0⋆x\right)\wedge \left(d_{2}\wedge d_{3}\right)\left(0⋆x\right)\\ \;=\left(d_{1}\left(0\right)⋆x\right)\wedge \left(d_{2}\left(0\right)⋆x\right)\\ \;=\left(\left(d_{1}\left(0\right)⋆x\right)⋆\left(d_{2}\left(0\right)⋆x\right)\right)⋆\left((d_{2}\left(0\right)⋆x\right)\\ =d_{1}\left(0\right)⋆x \end{array}$$ (8)

From (7) and (8) we have (d _1_ ∧ d _2_) ∧ d _3_)(x) = d _1_ ∧ (d _2_ ∧ d _3_)(x), ∀x ∈ X. Hence “∧” is associative.

Combining the above two lemmas we get following theorem.

Theorem 22. LDer(X) is a semigroup under the binary operation defined by (d _1_ ∧ d _2_)(x) = d _1_(x)∧d _2_(x) ∀x∈X, where d _1_,d _2_∈LDer(X).

Definition 16. Let RDer(X) be the set of all (l, r)-derivations on X. Define a binary operation “∧” on RDer(X) by (d _1_ ∧ d _2_)(x) = d _1_(x) ∧ d _2_(x) for all x ∈ X, where d _1_, d _2_ ∈ RDer(X).

Lemma 3. If d _1_ and d _2_ are (r, l)-derivations on X, then (d _1_∧d _2_) is also a (r, l)-derivation on X.

Proof. Let d _1_ and d _2_ are (r, l)-derivations on X. Then

$$\begin{array}{l} \left(d_{1}\wedge d_{2}\right)\left(x⋆y\right)=d_{1}\left(x⋆y\right)\wedge d_{2}\left(x⋆y\right)\\ \;=\left(x⋆d_{1}\left(y\right)\right)\wedge \left(x⋆d_{2}\left(y\right)\right)\\ \;=\left(\left(\left(x⋆d_{1}\left(y\right)\right)⋆\left(x⋆d_{2}\left(y\right)\right)\right)⋆\left(x⋆d_{2}\left(y\right)\right)\right)\\ \;=x⋆d_{1}\left(y\right) \end{array}$$ (9)

$$\begin{array}{l} x⋆\left(d_{1}\wedge d_{2}\right)\left(y\right)=x⋆\left((d_{1}\left(y\right)\wedge d_{2}\left(y\right)\right)\\ \;=x⋆\left(\left(d_{1}\left(y\right)⋆d_{2}\left(y\right)\right)⋆d_{2}\left(y\right)\right)\\ \;=x⋆d_{1}\left(y\right) \end{array}$$ (10)

From (9) and (10) we have (d _1_ ∧ d _2_)(x ⋆ y) = x ⋆ (d _1_ ∧ d _2_)(y). Hence, (d _1_ ∧ d _2_) (l, r)-derivation on X.

Lemma 4. The binary operation “∧” defined on RDer(X) is associative.

Proof. Let X be a PMS-algebra.

Let d _1_, d _2_, d _3_ be (r, l)-derivations on X.

Now

$$\begin{array}{l} \left(\left(d_{1}\wedge d_{2}\right)\wedge d_{3}\right)\left(x\right)=\left(\left(d_{1}\wedge d_{2}\right)\wedge d_{3}\right)\left(0⋆x\right)\\ \;=\left(d_{1}\wedge d_{2}\right)\left(0⋆x\right)\wedge d_{3}\left(0⋆x\right)\\ \;=\left(d_{1}\left(0\right)\wedge d_{2}\left(x\right)\right)\wedge \left(0⋆d_{3}\left(x\right)\right)\\ \;=\left(0⋆d_{1}\left(x\right)\right)⋆\left(d_{3}\left(x\right)⋆y)\right)⋆\left(d_{3}\left(x\right)⋆y\right)\\ \;=d_{1}\left(x\right)⋆y \end{array}$$ (11)

$$\begin{array}{l} \left(d_{1}\wedge \left(d_{2}\wedge d_{3}\right)\right)\left(x⋆y\right)=d_{1}\left(x⋆y\right)\wedge \left(d_{2}\wedge d_{3}\right)\left(x⋆y\right)\\ \;=\left(d_{1}\left(x\right)⋆y\right)\wedge \left(d_{2}\left(x\right)⋆y\right)\\ \;=\left(\left(d_{1}\left(x\right)⋆y\right)⋆\left(d_{2}\left(x\right)⋆y\right)\right)⋆\left((d_{2}\left(x\right)⋆y\right)\\ \;=d_{1}\left(x\right)⋆y \end{array}$$ (12)

From (11) and (12) we have (d _1_ ∧ d _2_) ∧ d _3_) = d _1_ ∧ (d _2_ ∧ d _3_). Combining the above two lemmas we get following theorem.

Theorem 23. RDer(X) is a semigroup under the binary composition defined by (d _1_ ∧ d _2_)(x) = d _1_(x) ∧ d _2_(x) ∀x ∈ X and d _1_, d _2_ ∈ RDer(X).

Lemma 5. Let (X; ⋆, 0) be a PMS-algebra and d _1_, d _2_ be (l, r)-derivations on X. Then the composition (d _1_ ◦ d _2_) is a (l, r)-derivation on X.

Proof. (d _1_ ◦ d _2_)(x⋆ y) = d _1_(d _2_(x⋆ y)) = d _1_(d _2_(x) ⋆ y) = d _1_(d _2_(x)) ⋆ y = (d _1_ ◦ d _2_)(x) ⋆ y. Hence, (d1 ◦ d2) is a (l, r)-derivation on X.

Lemma 6. Let (X; ⋆, 0) be a PMS-algebra and d _1_, d _2_ be (r, l)-derivations on X. Then the composition (d _1_ ◦ d _2_) is a (r, l)-derivation on X.

Proof. (d _1_ ◦ d _2_)(x⋆ y) = d _1_(d _2_(x⋆ y)) = d _1_(x⋆ d _2_(y)) = x⋆ d _1_(d _2_(y)) = x⋆ (d _1_ ◦ d _2_)(y). Hence, (d _1_ ◦ d _2_) is a (r, l)-derivation on X.

Theorem 24. Let (X; ⋆, 0) be a PMS-algebra and d _1_, d _2_ be derivations on X. Then the composition (d _1_ ◦ d _2_) is also a derivation on X.

Theorem 25. Let (X; ⋆, 0) be a PMS-algebra and d _1_, d _2_ be derivations on X. Then d _1_ ◦ d _2_ = d _2_ ◦ d _1_.

Proof. Given d _1_, d _2_ be derivation on X. Then d _1_, d _2_ are both (l, r) and (r, l)-derivations on X. Now

$$\begin{array}{l} \left(d_{1}◦d_{2}\right)\left(x\right)=\left(d_{1}◦d_{2}\right)\left(0⋆x)\right)\\ \;=d_{1}\left(d_{2}\left(0⋆x\right)\right)\\ \;=d_{1}\left(d_{2}\left(0\right)⋆x\right)\\ \;=d_{2}\left(0\right)⋆d_{1}\left(x\right) \end{array}$$ (13)

$$\begin{array}{l} \left(d_{2}◦d_{1}\right)\left(x\right)=\left(d_{2}◦d_{1}\right)\left(0⋆x\right)\\ \;=d_{2}\left(d_{1}\left(0⋆x\right)\right)\\ \;=d_{2}\left(0⋆d_{1}\left(x\right)\right)\\ \;=d_{2}\left(0\right)⋆d_{1}\left(x\right) \end{array}$$ (14)

From (13) and (14) we have (d _1_ ◦ d _2_)(x) = (d _2_ ◦ d _1_)(x). Therefore, d _1_ ◦ d _2_ = d _2_ ◦ d _1_.

Theorem 26. Let X be a PMS-algebra and d be a regular (l, r)-derivation on X. Define d ^2^(x) = d(d(x)) for all x ∈ X. Then d ^2^ = d.

Proof. Now

$$\begin{array}{l} d^{2}\left(x\right)=d\left(d\left(x\right)\right)\\ \;=d\left(d\left(0⋆x\right)\right)\\ \;=d(\left(d\left(0\right)⋆x\right)⋆\left(0⋆d\left(x\right)\right)\\ \;=d\left(\left(0⋆x\right)\wedge d\left(x\right)\right)\\ \;=d\left(x\wedge d\left(x\right)\right)\\ \;=d\left(\left(x⋆d\left(x\right)\right)⋆d\left(x\right)\right)\\ \;=d\left(\left(d\left(x\right)⋆d\left(x\right)\right)⋆x\right)\\ \;=d\left(0⋆x\right)\\ \;=d\left(x\right),\forall x\in X. \end{array}$$

Hence d ^2^ = d.

Definition 17. Let (X; ⋆, 0) be a PMS-algebra. Let d _1_, d _2_ be self-maps on X. We define (d _1_ ⋆ d _2_): X→ X as (d _1_ ⋆ d _2_)(x) = d _1_(x) ⋆ d _2_(x) ∀x ∈ X.

Theorem 27. Let (X; ⋆, 0) be a PMS-algebra and d _1_, d _2_ be derivations on X. Then d _1_ ⋆ d _2_ = d _2_ ⋆ d _1_.

Proof. Let x ∈ X.

$$\begin{array}{l} \left(d_{1}◦d_{2}\right)\left(x\right)=\left(d_{1}◦d_{2}\right)\left(0⋆x\right)\\ \;=d_{1}\left(d_{2}\left(0⋆x\right)\right)\\ \;=d_{1}\left(d_{2}\left(0\right)⋆x\right)\\ \;=d_{2}\left(0\right)⋆d_{1}\left(x\right)\\ \;=d_{2}\left(0\right)⋆d_{1}\left(x\right). \end{array}$$ (15)

$$\begin{array}{l} \left(d_{1}◦d_{2}\right)\left(x\right)=\left(d_{1}◦d_{2}\right)\left(0⋆x\right)\\ \;=d_{1}\left(d_{2}\left(0⋆x\right)\right)\\ \;=d_{1}\left(0⋆d_{2}\left(x\right)\right)\\ \;=d_{1}\left(0\right)⋆d_{2}\left(x\right)\\ \;=d_{1}\left(0\right)⋆d_{2}\left(x\right) \end{array}$$ (16)

From (15) and (16) we have d _1_(0) ⋆ d _2_(x) = d _2_(0) ⋆ d _1_(x) implies that (d _1_ ⋆ d _2_)(x) = (d _2_ ⋆ d _1_)(x), ∀x ∈ X.

Hence d _1_ ⋆ d _2_ = d _2_ ⋆ d _1_.

Definition 18. Let d a self-map on PMS-algebra of X. A PMS-ideal I of X is said to be d-invariant if d(I) ⊆ I, where d(I) = {d(x): x ∈ I}.

Theorem 28. Let d be a derivation on a PMS-algebra X. Then d is regular if and only if every PMS-ideal of X is d-invariant.

Proof. Suppose that d be a regular derivation.

Let y ∈ d(I). Then y = d(x) for some x ∈ I. Now y ⋆ x = d(x) ⋆ x = x ⋆ x = 0, since d is regular d(x) = x and hence y ⋆ x ∈ I as 0 ∈ I.

y = 0 ⋆ y = (x ⋆ x) ⋆ y = (y ⋆ x) ⋆ x ∈ I, since x ∈ I and y ⋆ x ∈ I. Thus, y ∈ I implies that d(I) ⊆ I.

Conversely, assume that every ideal of X is d-invariant, then d(0) ⊆ 0, and hence d(0) = 0.

Therefore, d is regular.

## 4. Generalized derivations on P MS-algebras

Definition 19. Let X be a PMS-algebra. A mapping D: X → X is called a generalized (l, r)-derivation on X if there exists a (l, r)-derivation d: X → X such that D(x ⋆ y) = (D(x) ⋆ y) ∧ (x ⋆ d(y), ∀ x, y ∈ X.

Definition 20. Let X be a PMS-algebra. A mapping D: X → X is called a generalized (r, l)-derivation on X if there exists a (r, l)-derivation d: X → X such that D(x ⋆ y) = (x ⋆ D(y)) ∧ (d(x) ⋆ y) for all x, y ∈ X.

Definition 21. Let X be a PMS-algebra. A mapping D: X → X is called a generalized derivation on X, if there exists a derivation d: X → X such that D is both a generalized (l, r)-derivation and a generalized (r, l)-derivation on X.

Example 7. Consider the PMS-algebra X = {0, 1, 2, 3, 4} as shown in Table 4:

**Table 4.** Generalized (r, l)-derivation on X.

⋆	*0*	*1*	*2*	*3*	* 4*
*0*	*0*	*1*	*2*	*3*	*4*
*1*	*4*	*0*	*1*	*2*	*3*
*2*	*3*	*4*	*0*	*1*	*2*
*3*	*2*	*3*	*4*	*0*	*1*
*4*	*1*	*2*	*3*	*4*	*0*

Define a self-map d: X → X by d(0) = 3, d(1) = 4, d(2) = 0, d(3) = 1, d(4) = 2.

Then d is a (r, l)-derivation on X. But d is not an (l, r)-derivation on X.

Define a map D: X → X by D(0) = 1, D(1) = 2, D(2) = 3, D(3) = 4, D(4) = 0.

Then D satisfies the equality, D(x ⋆ y) = (x ⋆ D(y)) ∧ (d(x) ⋆ y) for all x, y∈X. Thus D is a generalized (r, l)-derivation on X. But D is not a (l, r)-derivation on X.

Example 8. Let (X; ⋆, 0) be the PMS-algebra as shown in Cayley Table 5.

**Table 5.** Generalized derivation on X.

⋆	*0*	*1*	*2*	* 3*
*0*	*0*	*1*	*2*	*3*
*1*	*1*	*0*	*3*	*2*
*2*	*2*	*3*	*0*	*1*
*3*	*3*	*2*	*1*	*0*

A self-map d: X → X be defined by d(0) = 3, d(1) = 2, d(2) = 1, d(3) = 0.

Then d is a derivation on X.

Define a map D: X → X by D(0) = 2, D(1) = 3, D(2) = 0, D(3) = 1. D satisfies the equality, D(x ⋆ y) = (D(x) ⋆ y) ∧ (x ⋆ d(y)) for all x, y ∈ X and D(x ⋆ y) = (x ⋆ D(y)) ∧ (d(x) ⋆ y) for all x, y ∈ X. Thus D is a generalized derivation on X.

Theorem 29. Let D be a self-map on a PMS-algebra X. Then the following holds true.

1. If D is a generalized (r, l)-derivation on X and d be a regular (r, l)-derivation on X, then D(x) = D(x) ∧ x for all x ∈ X.
2. If D is a generalized (l, r)-derivation on X, then D(0) = 0 if and only if D(x) = x ∧ d(x) for all x ∈ X and for some regular (l, r)-derivation d of X.

Proof.

1. Let d be a regular derivation on X. Now
$$\begin{array}{l} D\left(x\right)=D\left(0⋆x\right)\\ \;=\left(0⋆D\left(x\right)\right)\wedge \left(d\left(0\right)⋆x\right)\\ \;=D\left(x\right)\wedge \left(0⋆x\right)\\ \;=D\left(x\right)\wedge x \end{array}$$
2. Suppose that D(0) = 0. Now
$$\begin{array}{l} D\left(x\right)=D\left(0⋆x\right)\\ \;=\left(D\left(0\right)⋆x\right)\wedge \left(0⋆d\left(x\right)\right)\\ \;=\left(0⋆x\right)\wedge d\left(x\right)\\ \;=x\wedge d\left(x\right) \end{array}$$
  Hence, D(x) = x∧d(x)
  Conversely, assume D(x) = x∧d(x) Now
$$\begin{array}{l} D\left(0\right)=0\wedge d\left(0\right)\\ \;=\left(0⋆d\left(0\right)\right)⋆d\left(0\right)\\ \;=\left(d\left(0\right)⋆d\left(0\right)\right)⋆0\\ \;=0⋆0\\ \;=0 \end{array}$$
  Hence D(0) = 0.

Theorem 30. Let D be a generalized (r, l)-derivation on a PMS-algebra X. Then

1. D(a) = a + D(0), ∀a ∈ X.
2. D(x + a) = x + D(a), ∀a, x ∈ X.
3. D(a + b) = D(a) + b = a + D(b), ∀a, b ∈ X.

Proof.

1.
$$\begin{array}{l} D\left(a\right)=D\left(\left(a⋆0\right)⋆0\right)\\ \;=\left(a⋆0\right)⋆D\left(0\right)\\ \;=\left(D\left(0\right)⋆0\right)⋆a\\ \;=a+D\left(0\right). \end{array}$$
Hence, D(a) = a + D(0).
2.
$$\begin{array}{l} D\left(x+a\right)=D\left(\left(a⋆0\right)⋆x\right)\\ \;=D\left(\left(x⋆0\right)⋆a\right)\\ \;=\left(x⋆0\right)⋆D\left(a\right)\\ \;=\left(\left(D\left(a\right)⋆0\right)⋆x\right)\\ \;=x+D\left(a\right) \end{array}$$
Hence, D(x + a) = x + D(a).
3. Since (X, +) is an abelian group, a + b = b + a, ∀a, b ∈ X. Now
  i.
$$\begin{array}{l} D\left(a\right)+b=D\left(a+b\right)\\ \;=D\left(b+a\right)\\ \;=D\left(b\right)+a \end{array}$$
Hence D(a + b) = D(a) + b = a + D(b), ∀a, b ∈ X.

Theorem 31. Let D be a generalized (l, r)-derivation on a PMS algebra X. Then

1. D(a)∈G(X) ∀a∈G(X).
2. D(a) = a ⋆ D(0) = a + D(0) ∀a∈X.
3. D(a + b) = D(a) + D(b) - D(0), ∀ a, b ∈ X.
4. D is the identity map on X if and only if D(0) = 0.

Proof.

1. Let a ∈ G(X), then a ⋆ 0 = a.
$$\begin{array}{l} D\left(a\right)=D\left(a⋆0\right)\\ \;=\left(D\left(a\right)⋆0\right)\wedge \left(a⋆d\left(0\right)\right)\\ \;=\left(\left(D\left(a\right)⋆0\right)⋆\left(a⋆d\left(0\right)\right)\right)⋆\left(a⋆d\left(0\right)\right)\\ \;=\left(\left(a⋆d\left(0\right)\right)⋆\left(a⋆d\left(0\right)\right)\right)⋆\left((D\left(a\right)⋆0\right)\\ =0⋆\left((D\left(a\right)⋆0\right)\\ =D\left(a\right)⋆0 \end{array}$$
Therefore, D(a)∈G(X).
2.
$$\begin{array}{l} D\left(a\right)=D\left(0⋆a\right)\\ \;=\left(D\left(0\right)⋆a\right)\wedge \left(0⋆d\left(a\right)\right)\\ \;=\left(\left(D\left(0\right)⋆a\right)⋆d\left(a\right)\right)\\ \;=\left(d\left(a\right)⋆d\left(a\right)\right)⋆\left(D\left(0\right)⋆a\right)\\ \;=D\left(0\right)⋆a \end{array}$$
Hence, D(a) = D(0) ⋆ a Again
$$\begin{array}{l} D\left(a\right)=D\left(0⋆a\right)\\ \;=D\left(0\right)⋆a\\ \;=D\left(0⋆0\right)⋆a\\ \;=\left(D\left(0\right)⋆0\right)⋆a\\ \;=D\left(0\right)+a \end{array}$$
Therefore, D(a) = D(0) ⋆ a = D(0) + a.
3.
$$\begin{array}{l} D\left(a+b\right)=a+b+D\left(0\right)\\ \;=a+D\left(0\right)+b+D\left(0\right)-D\left(0\right)\\ \;=D\left(a\right)+D\left(b\right)-D\left(0\right) \end{array}$$
Hence, D(a + b) = D(a) + D(b)-D(0).
4. Suppose D be an identity map on X. Then D(a) = a for all a∈X. Thus, D(0) = 0.
Conversely, assumed (0) = 0. Now
$$\begin{array}{l} D\left(a\right)=D\left(0⋆a\right)\\ \;=D\left(0\right)⋆a\\ \;=0⋆a\\ \;=a \end{array}$$
  Thus, D is an identity map on X.

## 5. Torsion-Free P MS-algebras

Definition 22. A PMS-algebra X is said to be torsion-free if x = 0 whenever x + x = 0, ∀ x ∈ X.

Example 9. Let (X, ⋆, 0) be the PMS-algebra as shown in Cayley Table 6.

**Table 6.** Torsion free PMS-algebra.

⋆	*0*	*1*	*2*	*3*	* 4*
*0*	*0*	*1*	*2*	*3*	*4*
*1*	*4*	*0*	*1*	*2*	*3*
*2*	*3*	*4*	*0*	*1*	*2*
*3*	*2*	*3*	*4*	*0*	*1*
*4*	*1*	*2*	*3*	*4*	*0*

0 + 0 = 0

1 + 1 = (1 ⋆ 0) ⋆ 1 = 4 ⋆ 1 = 2

2 + 2 = (2 ⋆ 0) ⋆ 2 = 3 ⋆ 2 = 4

3 + 3 = (3 ⋆ 0) ⋆ 3 = 2 ⋆ 3 = 1

4 + 4 = (4 ⋆ 0) ⋆ 4 = 1 ⋆ 4 = 3

Thus X is a Torsion-free PMS-algebra.

Theorem 32. Let X be a Torsion-free PMS-algebra and let D _1_ and D _2_ be two generalized derivations on X. If D _1_ ◦ D _2_ = 0 on X, then D _2_ = 0 on X.

Proof. Let x ∈ X, then x + x ∈ X.

$$\begin{array}{l} 0=\left(D_{1}◦D_{2}\right)\left(x+x\right)\\ \;=D_{1}\left(D_{2}\left(x+x\right)\right)\\ \;=D_{1}\left(0\right)+D_{2}\left(x+x\right)\\ \;=D_{1}\left(0\right)+D_{2}\left(x\right)+D_{2}\left(x\right)-D_{2}\left(0\right)\\ \;=D_{1}\left(0\right)-D_{2}\left(0\right)+D_{2}\left(x\right)+D_{2}\left(x\right)\\ \;=\left(D_{2}\left(0\right)⋆D_{1}\left(0\right)\right)+D_{2}\left(x\right)+D_{2}\left(x\right)\\ \;=\left(D_{1}\left(0\right)⋆0\right)⋆D_{2}\left(0\right)+D_{2}\left(x\right)+D_{2}\left(x\right)\\ \;=D_{1}\left(0\right)+D_{2}\left(0\right)+D_{2}\left(x\right)+D_{2}\left(x\right)\\ \;=D_{1}\left(D_{2}\left(0\right)\right)+D_{2}\left(x\right)+D_{2}\left(x\right)\\ \;=0+D_{2}\left(x\right)+D_{2}\left(x\right) \end{array}$$

Hence D _2_(x) + D _2_(x) = 0.

Since X is a Torsion-free, D _2_(x) = 0, ∀x ∈ X. Thus D _2_ = 0 on X.

Corollary 2. Let X be a Torsion-free PMS-algebra and D be a generalized derivation on X. If D ^2^ = 0 on X, then D = 0 on X.

Proof. Let x ∈ X, then x + x ∈ X.

$$\begin{array}{l} 0=D^{2}\left(x⋆x\right)\\ \;=D\left(D\left(x+x\right)\right)\\ \;=D\left(0\right)+D\left(x+x\right)\\ \;=D\left(0\right)+D\left(x\right)+D\left(x\right)-D\left(0\right) \end{array}$$

Hence D(x) + D(x) = 0.

Since X is torsion free D _2_(x) = 0, ∀ x ∈ X. Thus D _2_ = 0 on X

## 6. Conclusions

Derivation is a crucial research area in the field of algebraic structure in mathematics. The derivations on PMS-algebras were applied, and further properties of the derivations on PMS-algebras were discussed. The study focused on generalized derivations in PMS-algebras, including regular derivations, fixed set derivations, and composition of derivations. We demonstrated that the set of all derivations on a PMS-algebra X forms a semigroup under a suitable binary composition. The definitions and main results can be applied to other algebraic systems and to computational physics and computer sciences. Further research is needed to explore the application of these concepts in computer science for information processing, and derivations have also been employed in coding theory. This idea may extend to other algebraic structures.

## Ethics and consent

Ethical approval and consent were not required.

## Data Availability

No data are associated with this article.
