# Supplementary material for: Group Structures and Derivations on  PMS-algebras
Source: F1000Res. 2025 Jan 13;14:75. [Version 1] doi: 10.12688/f1000research.159712.1 (PMC12223485; doi:10.12688/f1000research.159712.1)
Supplement: Peer review history

# Reviewer report: Reviewer response for version 1

**DOI:** 10.5256/f1000research.175479.r393129

**Published:** 2025-07-02

**Authors:** Hemavathi P, Dr. Vinod Kumar R

**Affiliations:** 1 Mathematics, Rajalakshmi Engineering College, Thandalam/Chennai, Tamil Nadu, India; 2 Saveetha Institute of Medical and Technical Sciences (SIMATS), Chennai, Tamil Nadu, India

This paper investigates the concept of ideals within the framework of PMS-algebras—a class of algebraic structures that generalizes several well-known systems such as BCK-, BCI-, and TM-algebras. The authors define and explore various types of ideals (left, right, two-sided, bi-ideals, and interior ideals), providing structural results and demonstrating the interplay among them through propositions and examples

**Positive Aspects**

- The manuscript addresses an **important and underexplored topic** in algebraic systems, especially concerning the ideal structure of PMS-algebras.
- The **mathematical definitions are precise**, and the results are stated and proved rigorously.
- A **good balance of theory and examples** is maintained. The examples are well-chosen and help in clarifying the abstract notions.
- The discussion of **absorbing elements and identity-related properties** within the context of ideals is a welcome addition and contributes to a more complete understanding of PMS-algebra structures.

**Areas for Improvement**

1. **Abstract and Introduction**:
  - The abstract could better reflect the **core results and contributions** of the paper. It is currently too general and lacks specificity regarding the key findings.
  - A clearer **motivation for studying ideals in PMS-algebras** would enhance the introduction. For instance, how do these structures relate to or extend previous work in logic or algebra?
2. **Clarity and Readability**:
  - Some sentences are **awkwardly constructed** or overly formal. Simplifying and clarifying the language would improve accessibility, particularly for readers new to PMS-algebras.
  - Consider breaking down longer paragraphs, especially in the proof sections, for **better flow and readability**.
3. **Notational Consistency**:
  - Ensure all notations (especially for operations and mappings) are **defined consistently** and used uniformly across the manuscript.
  - A **glossary of notations** at the start or end of the paper would be beneficial.
4. **Structure and Formatting**:
  - Some **minor typographical issues** (e.g., inconsistent punctuation, misaligned equations) should be corrected for professional presentation.

**Minor Suggestions**

- Capitalization in section titles and definitions should be made consistent.
- Ensure that all cited references are correctly formatted and updated.
- It may be worth summarizing key results at the end of each section to improve digestibility.

**Recommendation:**

**Minor Revision**

 This is a promising and well-developed theoretical manuscript. With some refinements to the abstract, presentation, and organization, it will make a valuable contribution to the literature on PMS-algebras and generalized algebraic structures.

Is the work clearly and accurately presented and does it cite the current literature?

Yes

If applicable, is the statistical analysis and its interpretation appropriate?

Not applicable

Are all the source data underlying the results available to ensure full reproducibility?

No source data required

Is the study design appropriate and is the work technically sound?

Yes

Are the conclusions drawn adequately supported by the results?

Yes

Are sufficient details of methods and analysis provided to allow replication by others?

Yes

Reviewer Expertise:

Fuzzy algebra, Neutrosophic Sets, Fuzzy Decision Making, Topology

We confirm that we have read this submission and believe that we have an appropriate level of expertise to confirm that it is of an acceptable scientific standard.

# Reviewer report: Reviewer response for version 1

**DOI:** 10.5256/f1000research.175479.r369263

**Published:** 2025-03-21

**Authors:** Mezgebu Manmekto Gereme

**Affiliations:** 1 Department of Mathematics, Debark University, Debark, Amhara, Ethiopia

The article, “Group Structure and Derivation on PMS-algebras,” presents a compelling investigation. Using mathematical techniques, the researchers analyzed the properties and behavior of derivations within PMS-algebras. Specifically, they demonstrated that the set of all derivations associated with the defined binary operations forms a semi-group. This analysis offers a comprehensive understanding of generalized derivations, d-invariant ideals, fixed sets, and torsion-free PMS-algebras within this algebraic framework.

 Overall, the article effectively explores various aspects of torsion-free and generalized PMS-algebras, as well as the group structure and derivations on PMS-algebras. Consequently, this manuscript makes a significant contribution to the study of derivations on PMS-algebras. While the article is rich in proven results, the introduction could be strengthened by explicitly stating the motivation for this research. However, the authors adequately highlighted the study's importance and main contributions in the conclusion part of the manuscript. Therefore, I approved the acceptance for indexing in the journal.

Is the work clearly and accurately presented and does it cite the current literature?

Yes

If applicable, is the statistical analysis and its interpretation appropriate?

Not applicable

Are all the source data underlying the results available to ensure full reproducibility?

No source data required

Is the study design appropriate and is the work technically sound?

Yes

Are the conclusions drawn adequately supported by the results?

Yes

Are sufficient details of methods and analysis provided to allow replication by others?

Yes

Reviewer Expertise:

Fuzzy algebras, algebraic coding theory, Abstract algebras, Fuzzy coding theory, cryptography and fuzzy modeling.

I confirm that I have read this submission and believe that I have an appropriate level of expertise to confirm that it is of an acceptable scientific standard.

# Response

**Published:** 2025-05-26

**Authors:** Nibret Melese Kassahun

**Affiliations:** Mathematics, Bahir Dar University College of Science, Bahir Dar, Amhara, Ethiopia

We have read the comments made by the reviewer and we have been accepted all of the comments. The comments and suggestions are very constructive which motivates our next works.

 Thank yu

 Nibret Melese Kassahun

 nibret.melese2010@gmail.com
